# Meta-sGWAS: Integrating brain spatial interactions to uncover genetic variants in Bipolar Disorder

**DOI:** 10.21203/rs.3.rs-5791934/v1

**Published:** 2025-10-31

**Authors:** Wentao Li, Aoqi Wang, Yanfei Wang, Hongbo Bao, Kai Chen, Qianqian Song, Jia Wu, Xiaobo Zhou

**Affiliations:** 1 McWilliams School of Biomedical Informatics, University of Texas Health Science Center at Houston, 7000 Fanin St, Houston, 77030, TX, USA.; 2 Department of Imaging Physics, The University of Texas MD Anderson Cancer Center, 1515 Holcombe Blvd, Houston, 77030, TX, USA.; 3 Lung Cancer Center and West China Biomedical Big Data Centre, West China Hospital, Chengdu, 610041, China.; 4 Department of Health Outcomes & Biomedical Informatics, University of Florida, 1889 Museum Rd, Gainesville, 32611, FL, USA.; 5 Department of Neurosurgery, Beijing Tiantan Hospital, Capital Medical University, 119 Western Road of the Southern 4th Ring Road, Beijing, 100070, China.; 6 Department of Neurosurgery, Harbin Medical University Cancer Hospital, 150 Haping Rd, Harbin, 150001, China.; 7 Department of Biostatistics, The University of Texas MD Anderson Cancer Center, 1515 Holcombe Blvd, Houston, 77030, TX, USA.

**Keywords:** spatial GWAS, brain imaging, multimodal, mendelian randomization

## Abstract

Bipolar Disorder (BD) is influenced by both genetic factors and structural changes in brain regions, along with the impact of external environmental factors on brain network interactions. Conventional genome-wide association studies (GWAS) have identified genetic variants linked to BD but often overlook the spatial interactions and correlations in the brain. In this study, we introduced Spatial Genome-Wide Association Studies with Meta-analysis (Meta-sGWAS), a novel approach to decode the brain areas’ interaction patterns and related genetic underpinnings. Based on the genomic and brain MRI datasets from Adolescent Brain Cognitive Development (ABCD) and the UK Biobank (UKB), Meta-sGWAS first revealed significant associations between brain ROIs and BD, as well as the interactions among these ROIs. Notably, the “Right-G_subcallosal” ($P=9.9\times 10^{-9}$) and “Left-G_and_S_paracentral” ($P=3.6\times 10^{-8}$) were linked to disrupted cognitive and emotional processing in BD. Abnormal connectivity in the right orbitofrontal cortex further highlighted its role in BD-related emotional dysregulation. Meta-sGWAS then incorporated brain ROI interactions to identify genomic traits in a more robust way. From both ABCD and UKB datasets, Meta-sGWAS identified 6 SNPs related to brain-BD interactions. Two SNPs *rs1752582* and *rs1777305* within the novel identified *SVIL* gene, linked to brain development, suggest a potential brain-BD connection. Other two SNPs *rs12290811* and *rs113779084* were found highly enriched in BD-related cells, residing in *TENM4* and *THSD7A* genes, confirming their roles in inhibitory neurons-BD interaction. In summary, Meta-sGWAS integrates brain spatial interactions into genetic association studies, enhancing the accuracy of identifying significant genetic variants and elucidating their interactions. This approach provides valuable insights and potential biomarkers for understanding the brain mechanisms underlying BD pathogenesis.

## 1 Introduction

As one of the top leading causes of disability worldwide [1], Bipolar Disorder (BD) is a chronic illness characterized by shifts between mania or hypomania and depression. Evidence from population-based studies [2–4] suggests that the etiology of BD is multi-factorial, involving a complex interplay between genetic predisposition and environmental influences. These factors affect both volumetric and systematic variations in brain structure and function, which are closely associated with heritable psychiatric disorders such as BD. Recent research has focused on uncovering the systematic relations between BD, genetic variants, and brain regions of interest (ROIs) [5, 6]. For example, Matsuo et al. [7], suggest that morphometric brain abnormalities of the anterior-limbic neural substrate are associated with family history of BD. Other studies [8] showed gray matter reductions and white matter integrity deficits would also cause the vulnerability of developing BD. And this abnormality in brain ROIs also has a strong correlation to genetic traits, Genome-wide association studies (GWAS) studies suggested a significant association between these brain ROIs and genetic variants, e.g., bilateral putamen and rs1800562 ($P=6.6\times 10^{-20}$), caudate nucleus and rs4428180 ($P=2.23\times 10^{-22}$), pallidum and rs35469695 ($P=2.22\times 10^{-12}$), etc [6].

Previous GWAS studies have identified SNP loci associated with BD by either treating brain regions as independent entities [9] or considering the brain as a single, mixed functional unit based on measures such as cortical thickness, surface area, volume, and gyrification [10]. However, these studies didn’t take into account the spatial heterogeneity of the brain, limiting the insights into the spatial organization among distinct brain regions. This oversight may introduce bias, reducing the robustness and credibility of GWAS findings [11], leading to the limitation of p-value inflation and confounding effects bias [12–15]. Moreover, recent studies have highlighted spatial patterns of gene expression within tissues, suggesting that SNPs may exhibit spatial interactions across different brain regions. This spatial variability within complex brain ROI activity networks contributes to individual differences that significantly influence disease susceptibility [16, 17]. Therefore, integrating genetic data with spatial brain information is essential for a more comprehensive understanding of how genetic factors and brain activities collectively impact BD development.

**Fig. 1:**
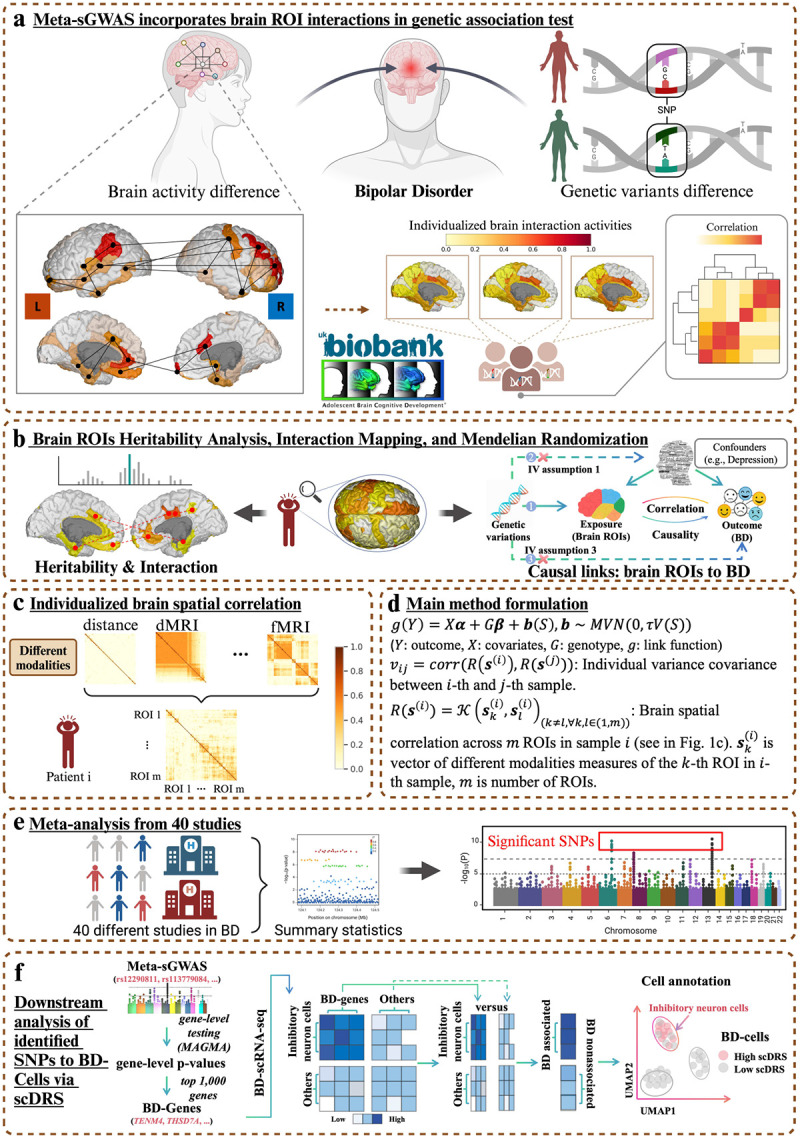
Meta-sGWAS: Identifying robust significant SNPs in BD combined with brain spatial interactions. a) Meta-sGWAS considers brain activity across different ROIs, incorporating both genetic variants and brain activity factors in studying the development of bipolar disorder (BD); b) Meta-sGWAS identifies BD-related significant brain ROIs and their correlations, followed by heritability analysis to uncover genetically influenced ROIs, and Mendelian Randomization to infer causal effects between these ROIs and BD. MR relies on three key assumptions: genetic variants (instrumental variables, IVs) must be strongly related to the exposure (ROIs), independent of confounders, and not directly linked to the outcome (BD) except through the exposure; c) By integrating data from multiple MRI modalities, sGWAS processes brain regional interactions and correlations via Gaussian Kernel function, creating a spatial-enriched kernel matrix from predefined ROIs. This spatial kernel matrix delves into the complex brain activity networks in genetic association testing. In contrast to conventional GWAS, which views the brain as a single entity and has proven less accurate due to this simplification, sGWAS provides a more accurate and robust method by addressing spatial correlation nuances in the brain activity network; d) The high-level modeling summary of sGWAS; e) Meta-analysis of 40 different studies on BD to further enhance the results of sGWAS; f) Our proposed method provides more robust and comprehensive insights into spatial-enriched genetic variant associations, using Meta-sGWAS-derived GWAS results with single-cell RNA-seq data to identify BD-associated cell types.

This study presents a comprehensive framework for understanding the genetic underpinnings of BD through a combination of imaging genetics, meta-analysis, and single-cell data integration (Fig. 1). First, brain activity differences associated with BD were analyzed using multimodal MRI data from datasets like ABCD and UK Biobank (Fig. 1a). Individualized brain interaction patterns were quantified, generating brain-specific spatial kernel matrices to link regional brain features (ROIs) to genetic variation (Fig. 1b). These features were further analyzed for heritability and their causal relationships with BD through Mendelian randomization (MR), uncovering critical brain regions implicated in BD pathogenesis (Fig. 1c). Spatial-interaction enriched GWAS was then performed with all ROI-derived features, enabling the identification of BD-associated SNPs (Fig. 1d). The approach was validated through meta-analysis across 40 BD studies (Fig. 1e), demonstrating its robustness and sensitivity in detecting SNPs with low genetic effects. Finally, downstream integration with single-cell RNA-seq data established a multi-scale link between SNPs, genes, cells, and BD, identifying inhibitory neurons as a key cell type associated with BD-related genetic signals (Fig. 1f). This workflow highlights the power of integrating multimodal data to unravel the complex biology of psychiatric disorders.

## 2 Results

We first benchmark our Meta-sGWAS framework and demonstrate its robust performance by outperforming conventional GWAS methods. Then, Meta-sGWAS was applied to real-world datasets, mapping brain measurements from the Adolescent Brain Cognitive Development (ABCD) and UK Biobank (UKB) datasets onto 148 ROIs using the Destrieux Atlas.

Meta-sGWAS revealed significant associations were observed between specific ROIs and BD. Also, brain interactions among ROIs indicated that these regions do not function independently. Then the heritability analysis results demonstrated substantial genetic contributions to these ROIs, and Mendelian randomization identified several significant causal relationships between ROIs and BD. Our novel method identified 15 significant single nucleotide polymorphisms (SNPs) in the ABCD dataset and 66 in the UKB dataset at the stringent threshold of $P<5\times 10^{-8}$. At a more relaxed threshold of $P<1\times 10^{-5}$, 104 SNPs were identified in the ABCD dataset and 235 in the UKB dataset. Then the downstream Single-cell transcriptomic analysis using scDRS validated the SNPs identified by Meta-sGWAS. This analysis linked the SNPs to specific cell types and diseases through gene expression enrichment scores, confirming the biological relevance of these findings.

### Evaluation of Meta-sGWAS framework

We first benchmarked our proposed Meta-sGWAS using synthetic data, mimicking a cohort of 1,000 individuals with 100 generated genotypes (20 of which are causal), 6 non-genetic covariate effects, and 100 ROIs in the brain. The primary objective of this experiment is to assess and compare the performance of our proposed Meta-sGWAS against conventional GWAS approaches. We evaluated our model using synthetic data generated with PhenotypeSimulator, a tool that integrates genetic and non-genetic components via linear mixed models (LMMs) for benchmarking. To simulate brain interaction patterns, we introduced individualized brain activity profiles sampled from a normal distribution ($\phi_{i}$) and constructed a 1,000-by-1,000 brain interaction correlation matrix using Pearson coefficients. Heatmaps (Supplementary Fig. S1) illustrate examples of these individualized patterns, aligning with advanced methods for analyzing complex brain traits.

The comparative experiment between Meta-sGWAS and conventional GWAS was repeated 50 times under different total SNP effect settings, representing the contribution of genetic variants directly influencing the phenotype. In this setup, the maximum total SNP effect was set to 0.8, with the remaining 0.2 accounting for random effects to simulate environmental influences on the phenotype. The performance assessment of each method was quantified by measuring the Area Under the Curve (AUC) of the Receiver Operating Characteristic (ROC) curve for predicting causal SNPs using both approaches.

**Fig. 2:**
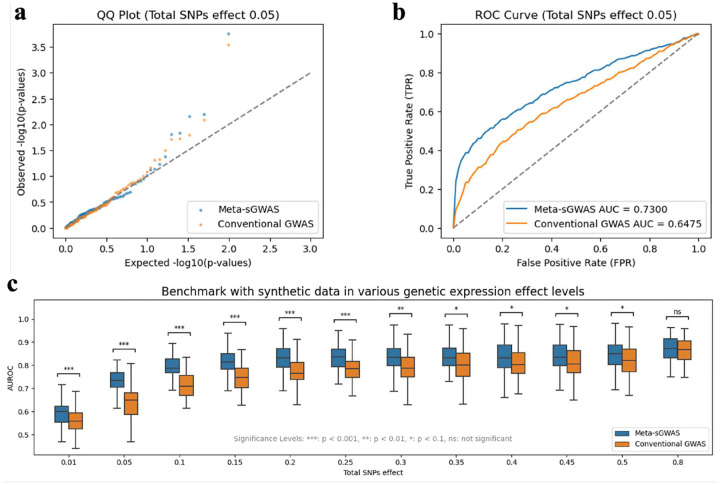
Synthetic data results. a) QQ-plot of P-values of conventional GWAS and Meta-sGWAS in synthetic data under total SNPs effect as 0.05; b) ROC curves of significant SNPs being identified in conventional GWAS and Meta-sGWAS under total SNPs effect as 0.05; c) Box plot comparing the AUROC of significant SNP identification using Meta-sGWAS versus conventional GWAS methods, with each box representing 50 independently generated random experiments. The “Total SNPs effect” metric reflects the impact of causal genetic variants on the disease, where a higher ratio indicates a stronger causal relationship.

The results showed that Meta-sGWAS consistently outperformed Conventional GWAS across 50 repeated experiments in all total SNP effect settings (Fig. 2c). The advantage of Meta-sGWAS was particularly significant at lower total SNP effect levels. As the total SNP effect ratio increased, the performance difference between Meta-sGWAS and Conventional GWAS narrowed. This trend is logical, as higher genetic contributions to the phenotype can be more easily detected by simpler conventional GWAS methods. Notably, the largest performance gap occurred at a total SNP effect of 0.05, where Meta-sGWAS achieved a higher mean ROC AUC of 0.73 compared to 0.65 for conventional GWAS (Fig. 2a and b).

### Meta-sGWAS reveals Brain ROIs that are associated with BD have complex interactions

**Fig. 3:**
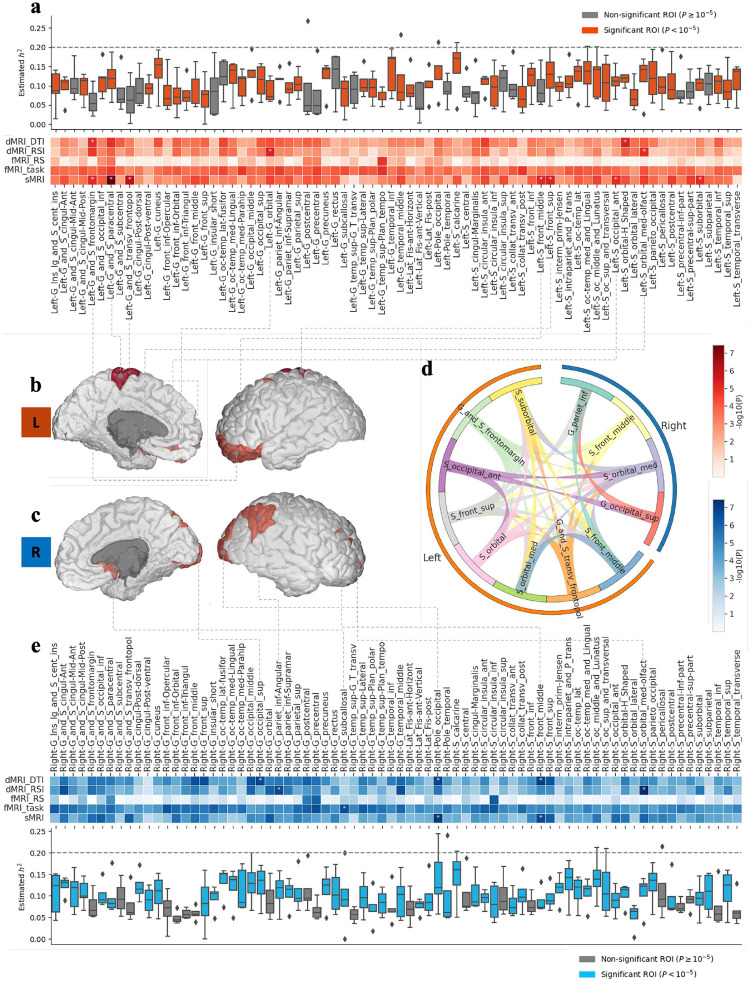
Brain ROIs association with BD in ABCD dataset. Brain ROI association and heritability analysis of the whole brain in the ABCD dataset highlighted significant brain areas with threshold $P<10^{-5}$ in asteroids (*). a) Estimated heritability (y-axis) of 74 ROIs in the left globe analyzed (n=8,848 subjects, see Method section for heritability calculation details), P-values < 10^−5^ are colored in orange and Heatmap of the t-test P-values in the left brain in 74 regions; b) Left hemisphere inner and outer cortical and subcortical brain anatomy with significant areas highlighted; c) Right hemisphere inner and outer cortical and subcortical brain anatomy with significant areas highlighted; d) Correlation among the 12 out of 16 significant ROIs ($P<10^{-5}$) with correlation values exceeding 0.6; e) Heatmap of the t-test P-values in the right brain in 74 regions and Estimated heritability (y-axis) of 74 ROIs in the right globe analyzed (n=8,848 subjects, see Method section for heritability calculation details), P-values < 10^−5^ Fre colored in blue.

Using real-world datasets, we investigated the connections between the human brain and psychiatric activity in BD. Meta-sGWAS performed two-sided t-tests on 148 brain ROIs mapped using the Destrieux Atlas across two datasets: the Adolescent Brain Cognitive Development (ABCD) dataset (case = 1,514; control = 7,831) and the UK Biobank (UKB) dataset (case = 116; control = 11,093). Our analysis identified 16 ROIs significantly associated with BD at a P-value threshold of 10^−5^. Notably, the “Right-G_subcallosal,” located in the right nucleus accumbens (fMRI-task, $P=9.9\times 10^{-9}$), has been implicated in integrating cognitive and emotional information, contributing to conditions such as addiction, depression, and schizophrenia. Similarly, the “Left-G_and_S_paracentral” (sMRI, $P=3.6\times 10^{-8}$) has been linked to disrupted emotional and cognitive processing observed during depressive episodes in BD. Additionally, the right orbitofrontal cortex (OFC) showed abnormal functional connectivity, including increased connectivity with the insula and ventrolateral prefrontal cortex, potentially underlying the emotional and cognitive dysregulation characteristic of BD. Heatmaps of the t-test results (heatmaps in Fig. 3a, e) visually depict these associations, with darker colors indicating lower P-values and stronger correlations between specific ROIs and BD. For clarity, we annotated and mapped the significant ROIs ($P<10^{-5}$) onto brain anatomy figures, providing a comprehensive visualization of these findings.

The further investigation of the correlations among brain ROIs found that these regions do not impact the development of BD independently. To uncover underlying patterns, Meta-sGWAS performed hierarchical clustering using data from five different MRI modalities of BD patients in the ABCD dataset. The clustering analysis, visualized in Supplementary Fig. S2 with a z-score heatmap, revealed a complex network of interactions across ROIs in both hemispheres of the brain, emphasizing their interconnected roles in BD. Pearson correlation analysis further demonstrated significant variability in connectivity across the 148 ROIs, particularly among the 16 regions identified as significant ($P<10^{-5}$). Specifically, 12 of these regions—including Frontal Lobe areas (orbitofrontal, middle, and superior frontal regions), the Parietal Lobe (angular gyrus), and Occipital Lobe areas (anterior and superior occipital regions)—exhibited Pearson correlation values exceeding 0.6 (Fig. 3d). The finding underscores the complex interplay among brain regions involved in emotion regulation, cognitive control, sensory processing, and social cognition, highlighting their collective contribution to BD pathophysiology.

### Meta-sGWAS reveals significant heritability of Occipital Lobe areas in BD

To investigate the correlation between genetic variants and brain ROIs, Meta-sGWAS conducted a multi-trait genome-wide association study (GWAS) to assess SNP heritability across various MRI modalities. The boxplots in Fig. 3a and e present the estimated SNP heritability ($h^{2}$) for all 148 ROIs, analyzed using five different MRI modalities, and whether these heritability estimates are significantly different from zero with a stringent P-value threshold of 10^−5^. The results (supplementary S1 Table) indicate that 106 out of 148 ROIs exhibit significant heritability in dMRI_DTI, 117 in dMRI_RSI, 38 in fMRI_RS, 140 in fMRI_task, and 126 in sMRI, demonstrating that sMRI is the most heritable modality among all.

The heritability analysis of significant ROIs with heritability scores greater than 0.2 aligns with BD-related ROIs, emphasizing that these brain regions are notably heritable. For instance, the temporal lobe region (“G_temporal_middle”), which is involved in language and semantic memory processing, has shown significant heritability, underlining the significant role of genetic factors in its variability. Also, the occipital region (“Pole_occipital”), a critical area for visual processing as part of the primary visual cortex, also displays high heritability, suggesting that the visual processing areas are greatly influenced by genetic components. Moreover, the pericallosal sulcus (“S_pericallosal”) could provide insights into interhemispheric communication and related cognitive functions.

### Meta-sGWAS reveals causal relationships between brain ROIs and BD

**Fig. 4:**
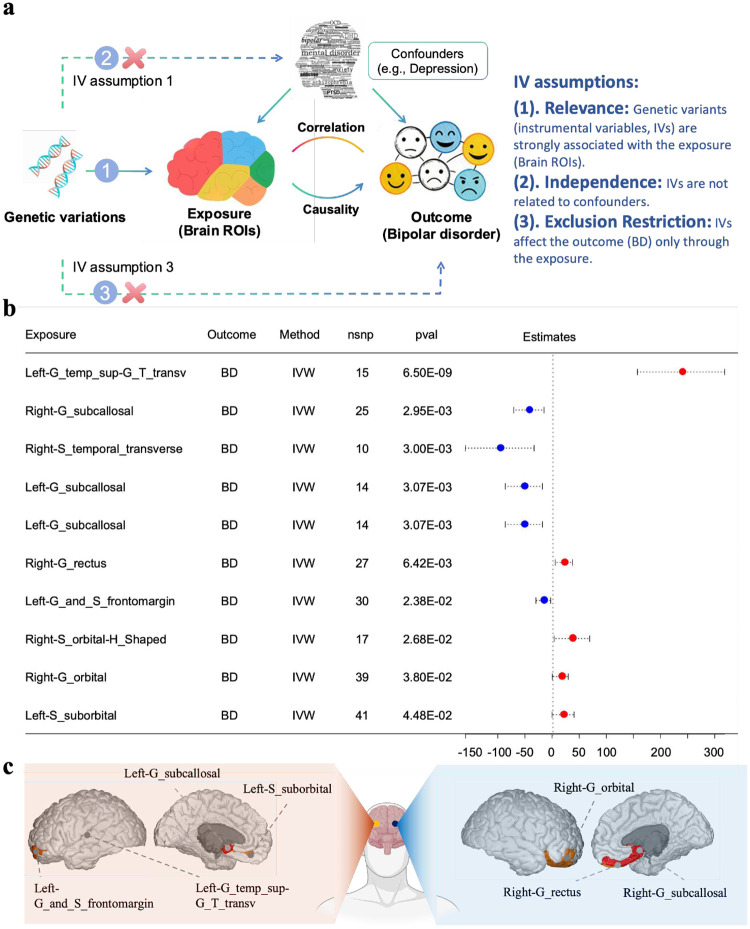
Mendelian Randomization (MR) Analysis of Brain ROIs and BD. a) MR framework illustrating the three core instrumental variable (IV) assumptions: (1) genetic variants (IVs) are strongly associated with the exposure (brain ROIs); (2) IVs are independent of confounders (e.g., depression); and (3) IVs affect the outcome (BD) only through the exposure. b) Summary of MR results for significant ROIs, showing causal effect estimates and confidence intervals. For example, “Left-G_temp_sup-G_T_transv” demonstrates a strong positive causal association ($p=6.57\times 10^{-9}$), while regions such as “Right-G_subcallosal” and “Right-S_temporal_transverse” exhibit negative causal associations. c) Anatomical visualization of significant ROIs across the brain, highlighting key regions in the left and right hemispheres, including “Left-G_temp_sup-G_T_transv,” “Left-G_and_S_frontomargin,” “Right-G_rectus,” and “Right-G_subcallosal”.

To investigate the relationship between brain ROIs and BD, we employed genetic correlation analysis and Mendelian Randomization (Fig. 4). We preliminarily applied linkage disequilibrium score regression (LDSC) to evaluate the genetic correlation between structural changes in different brain regions and BD. Genetic correlation analysis identified statistical associations between ROIs and BD, while MR assessed the causal effects of these ROIs on BD. As shown in Fig. 4a, the MR framework relies on three instrumental variable (IV) assumptions: (1) genetic variants (IVs) must be strongly associated with the exposure (ROIs); (2) IVs must be independent of confounders such as depression; and (3) IVs must affect the outcome (BD) only through the exposure. This framework distinguishes causation from correlation, providing insights into the causal roles of brain structural changes in BD.

Among the 148 ROIs analyzed, genetic correlation identified 14 regions with statistically significant associations with BD (P<0.05). These findings are illustrated in Fig. 4b, which displays the causal effect estimates and confidence intervals obtained using the Inverse Variance Weighted (IVW) method. For instance, “Left-G_temp_supG_T_transv” demonstrated a strong positive causal association ($P=6.57\times 10^{-9}$), suggesting that increased structural intensity in this region is associated with a higher risk of BD. Conversely, regions such as “Right-G_subcallosal” ($P=2.95\times 10^{-3}$) and “Right-S_temporal_transverse” ($P=3\times 10^{-3}$) showed significant negative causal associations, indicating that structural changes in these areas may reduce BD risk. The anatomical locations of these significant ROIs are visualized in Fig. 4c, highlighting key cortical regions in both the left and right hemispheres. Notable regions include “Left-G_temp_sup-G_T_transv,” “Left-G_and_S_frontomargin,” “Right-G_rectus,” and “Right-G_subcallosal.” This integrated analysis underscores the complex interactions between brain anatomy and BD, providing a deeper understanding of how specific ROIs contribute to BD pathogenesis.

### Individualized brain regional interactions are unique

Meta-sGWAS also highlights that each individual exhibits a unique interaction network of connectivity patterns across the 148 brain ROIs. Supplementary Fig. S3a and b present chord diagrams illustrating the interactions between the left and right hemispheres across 148 ROIs for the two samples: a BD patient and a non-BD patient from ABCD dataset. These regional brain connections were computed using a Gaussian kernel function in 5 different MRI modalities and the ROI distance, as described in the Methods section. The comparison highlights notable differences in brain connectivity patterns between the two individuals, with the BD patient in the selected sample exhibiting more active brain ROIs communication compared with another selected non-BD sample (Supplementary Fig. S3b). To quantify the similarity in brain connection patterns across individuals, we defined a variance-covariance matrix, utilizing correlation tests on the brain connection maps. For all 9,345 samples in the ABCD dataset, we performed pairwise Pearson correlation to assess the similarity of brain connectivity patterns. Supplementary Fig. S3c provides a snapshot of the correlation heatmap for 30 randomly selected samples from the cohort.

### Significant genetic variants identified with Meta-sGWAS in Bipolar Disorder from ABCD and UKB datasets

We implemented the Meta-sGWAS framework on both the ABCD and UKB datasets to estimate the association between BD status and the SNPs, considering individualized interaction patterns across 148 brain ROIs. The ABCD dataset incorporated five MRI modalities, while the UKB dataset utilized a single modality (sMRI). To account for multiple comparisons and control for correlations between genetic variants and BD, we used age, sex, gender, and the top 10 principal components as covariates to account for population stratification and applied a stringent P-value threshold of 5 × 10^−8^ to filter significant SNPs.

**Fig. 5:**
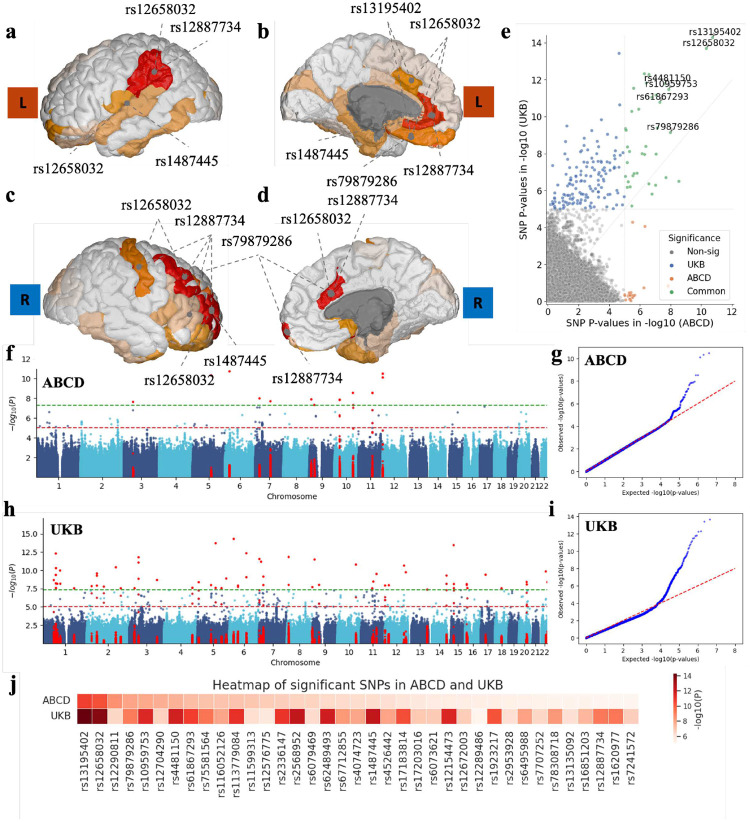
Meta-sGWAS identified significant genetic variants in both ABCD and UKB datasets. a)-d) SNPs association map in the cortical outer left hemisphere, cortical inner left hemisphere, cortical outer right hemisphere, and cortical inner right hemisphere (when multiple SNPs pointed to the same region, the color indicates the most significant P-values of the SNPs); e) Scatter plot of P-values from the Meta-sGWAS analysis comparing the ABCD and UKB datasets, with a significance threshold of < 1 × 10^−5^. Different colors indicate whether SNPs are significant in both datasets, only in the ABCD dataset, or only in the UKB dataset; f) Manhattan plot of Meta-sGWAS in the ABCD dataset, green dash line is < 5 × 10^−8^ and red dash line is < 1 × 10^−5^; g) QQ-plot of Meta-sGWAS results of 3, 948, 476 SNPs in the ABCD dataset; h) Manhattan plot of Meta-sGWAS in the UKB dataset, green dash line is < 5 × 10^−8^ and red dash line is < 1 × 10^−5^; i) QQ-plot of Meta-sGWAS results of 4, 323, 319 SNPs in the UKB dataset; j) Heatmap comparison of all significant SNPs identified in the ABCD and UKB datasets with threshold < 5 × 10^−8^.

Meta-sGWAS identified 15 SNPs with P-values below 5 × 10^−8^ in the ABCD cohort. A Manhattan plot (Fig. 5f) and QQ plot (Fig. 5g) summarize the genome-wide scans of 3,948,476 SNPs in the ABCD dataset. For example, *rs12658032* exhibited strong associations with various brain regions, including the right gyrus and sulcus of the anterior mid-cingulate cortex ($P=2\times 10^{-6}$), left gyrus and sulcus of the anterior cingulate cortex ($P=1.7\times 10^{-5}$), and the left sulcus of the medial orbital-olfactory cortex ($P=2.1\times 10^{-5}$). These regions are linked to emotional regulation and cognitive processing. In the UKB dataset, we identified 66 SNPs with P-values less than $P<5\times 10^{-8}$ and 232 SNPs below 10^−5^ from 4,323,319 SNPs, see the Manhattan plot (Fig. 5h) and QQ plot (Fig. 5i) results of UKB dataset. Notably, SNPs such as *rs1752582* and *rs1777305*, located in the *SVIL* gene, were identified. While these SNPs do not directly associate with brain ROIs, they play roles in neuronal maturation through LSD1+8a-mediated H3K9 demethylation and have been implicated in schizophrenia and brain-related traits such as aortic distensibility.

A scatter plot (Fig. 5e) compares the P-values from Meta-sGWAS analyses in the ABCD and UKB cohorts, and a heatmap (Fig. 5j) highlights significant SNPs shared between the two cohorts $\left(P<10^{-5}\right)$. The UKB cohort, consisting of older individuals, revealed more significant SNPs compared to the younger ABCD cohort, suggesting that BD-associated genetic factors may have a stronger influence in older populations. For example, we identified the SNP *rs56361249* ($P=1.41\times 10^{-7}$ in UKB, P=0.007 in ABCD) and *rs1518367* ($P=5.73\times 10^{-6}$ in UKB, P=0.05in ABCD), located in the *LMAN2L* gene and *PLCL1* gene respectively, which have been shown to affect the molecular aging rate or Hematopoietic stem cells which are essential for the lifelong maintenance of the blood system. However, some SNPs remained significant across age groups, suggesting consistent genetic risk factors throughout life.

Notably, six highly significant SNPs ($P<5\times 10^{-8}$) are found in both datasets (Supplementary Table S1). SNPs such as *rs13195402*, *rs12658032*, and *rs12290811* were consistently identified across both the ABCD and UKB datasets. These SNPs are also strongly associated with different brain regions (Fig. 5a–d), indicating their potential involvement in key biological pathways related to Bipolar Disorder. These shared findings lay the groundwork for further investigation through downstream single-cell analyses to reveal the underlying molecular mechanisms of the disorder.

### SNPs enriched in BD cells

**Fig. 6:**
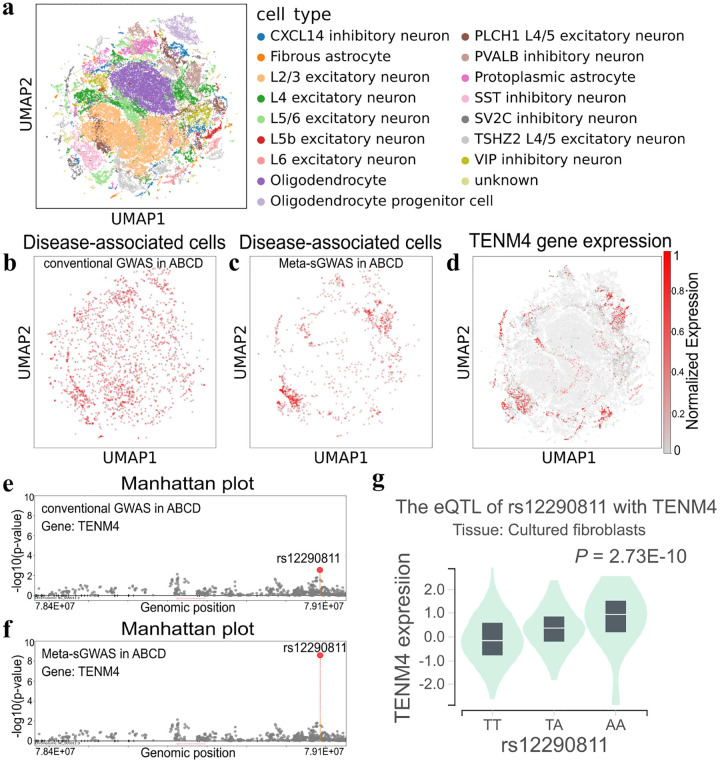
Downstream Single Cell Analysis of Spatial GWAS. a) A completely annotated single-cell transcriptomic map of the human BD disease from data generated using scRNA-seq applied to 24 human samples reported in SZBDMulti-Seq dataset and visualized using a UMAP plot; b) Employing the GWAS analysis results of BD from the ABCD database, the scDRS was utilized to assess the genome-wide genetic risk of each cell, and the disease-related cells of BD were marked with red dots (P<0.05, the one-sided Monte Carlo test); c) After correcting the GWAS results of BD from the ABCD database using the meta-sGWAS method, scDRS was calculated and BD related cells were labeled; d) By calculating the Pearson correlation between gene expression and revised scDRS score in each cell, it was found that *TENM4* gene was strongly correlated with scDRS (correlation coefficient was 0.34, p less than 0.001), and the UMAP showed the gene expression; e) A Manhattan plot of SNPs on the *TENM4* gene is shown, displaying the GWAS results prior to applying the Meta-sGWAS method; f) A Manhattan plot of SNPs on the *TENM4* gene is shown, displaying the results after applying the Meta-sGWAS method. And the P-value of rs12290811 has been revised from the original 5.27 × 10^−3^ to 2.87 × 10^−9^; g) The eQTL direction of the rs12290811 with *TENM4* in Cultured fibroblasts cells from the GTEx database. The gene expression implicates variation T to A of rs12290811, which might significantly enhance the gene expression of *TENM4*. eQTL, expression quantitative trait locus.

To further interpret the BD-associated SNPs identified by Meta-sGWAS, we conducted single-cell analysis using scRNA-seq data from 24 Bipolar Disorder samples in the SZBDMulti-Seq dataset. Cell types were annotated with the CELLiD R package (Fig. 6a). To evaluate the effectiveness of Meta-sGWAS, we compared heritability enrichment results from the ABCD and UKB datasets before and after applying Meta-sGWAS using scDRS to identify disease-related cells. Before Meta-sGWAS, BD-associated cells were randomly distributed across all cell types (Fig. 6b). After Meta-sGWAS correction in the ABCD dataset, scDRS revealed significant enrichment of BD-associated cells in specific inhibitory neuron types, including SST, PVALB, and SV2C neurons (Fig. 6c). Notably, SST inhibitory neurons play a crucial role in BD by maintaining the excitatory-inhibitory balance in the cerebral cortex, which is associated with symptom severity and episode frequency. This demonstrates the ability of Meta-sGWAS to enhance the biological relevance of SNP effect sizes.

Meta-sGWAS also identified a strong correlation between scDRS scores and *TENM4* gene expression (correlation coefficient = 0.34, P<0.001), with the expression pattern aligning with BD-associated cell distributions. The Meta-sGWAS analysis refined the P-value of the *rs12290811* variant within *TENM4* from 5.27 × 10^−3^ to 2.87 × 10^−9^, significantly strengthening its statistical association. The rs12290811 variant, a T-to-A mutation located in the intron of *TENM4*, was further investigated using eQTL analysis via the GTEx database. Results revealed that this mutation significantly upregulates *TENM4* expression ($P=2.73\times 10^{-10}$). Independent GWAS studies on BD also identified *rs12290811* as a risk variant ($P=1\times 10^{-9}$). These confirm that Meta-sGWAS not only identifies key risk SNPs but also corrects for false negatives, enhancing the overall accuracy and reliability of genetic associations.

Another significant SNP identified was *rs113779084* (Supplementary Fig. S6), located in the 5’ UTR of the *THSD7A* gene. Meta-sGWAS refined its P-value from 0.97 to 8.69 × 10^−8^, enhancing its statistical significance. Using the JASPAR database, we predicted that the G-to-A mutation in *rs113779084* reduces the binding affinity of the CTCF transcription factor, which regulates *THSD7A*. Functional validation with luciferase assays in SH-SY5Y and U251 cell lines confirmed that the A allele significantly increases reporter gene expression compared to the G allele, indicating a role in disrupting normal *THSD7A* regulation. Increased *THSD7A* expression in inhibitory neurons may impair their regulatory functions, exacerbating the excitatory-inhibitory imbalance and contributing to BD pathophysiology.

## 3 Discussion

BD exhibits varying prevalence across age groups [18, 19], populations [20], and sexes [21], with genetic backgrounds playing a significant role in these disparities [22]. Beyond genetics, structural and functional changes in specific brain regions are pivotal in BD progression. Integrating brain region-specific characteristics as covariates in GWAS analyses, alongside traditional factors like age, sex, and population structure, is essential for a comprehensive understanding of the interplay between genetic variants and brain alterations.

Meta-sGWAS analysis identified several significant genetic variants associated with BD in two large cohorts: ABCD (N=9,345) and UKB (N=11,093). We filtered the Meta-sGWAS results using a P-value threshold of 1 × 10^−5^, identifying 104 SNPs in the ABCD dataset and 235 SNPs in the UKB dataset, with 40 overlapping SNPs shared between them (see supplementary S2 Table).

To better understand the functional relevance of these SNPs, we used DisGeNET for annotation. DisGeNET aggregates data from expert-curated repositories, GWAS catalogs, animal models, and scientific literature, providing a comprehensive overview of relationships among SNPs, genes, and diseases. Through DisGeNET, we identified 332 studies linking these 40 SNPs to 45 distinct diseases (see supplementary S4 Table). A network diagram (Supplementary Fig. S4) visualizes the associations between the SNPs and diseases, showing that all 40 SNPs are annotated as related to Bipolar Disorder, with 14 SNPs uniquely associated with BD. This specificity suggests that these SNPs may play a unique role in the genetic risk for BD, providing important clues for identifying BD-specific genetic markers and understanding its pathophysiological mechanisms.

Particularly noteworthy is the identification of SNPs *rs1752582* and *rs1777305* within the SVIL gene, which had not previously been associated with BD but have been linked to aortic distensibility. This connection suggests a possible pathway between cardiovascular traits and psychiatric conditions, this topic also known as the brain-heart connection has been studied by Zhao et al. [23] Moreover, *rs12290811* (associated with Left Gyrus Subcallosal, P=0.004) is an intronic variant within the *TENM4* gene, which has been highlighted for its involvement in neuronal development [24]. Studies have demonstrated its role in neural patterning, axon guidance, and synaptic plasticity, critical processes in brain development and function. Moreover, large-scale genome-wide association studies (GWAS) have identified *rs12290811* as significantly associated with bipolar disorder, reinforcing its potential as a key genetic factor in the disorder’s etiology. Meta-sGWAS also identified rs13195402 and *rs12658032*, which are located in non-coding regions. However, a recent study showed that non-coding variants can “turn on” and “turn off” genes that code for proteins. This study reveals that Non-coding variants can enact an ‘environment-dependent’ function: they only become active when specific cellular pathways are activated. In other words, these genetic variants exert their on-off effect on proteins only when brain cells respond to stimuli.

Although the Meta-sGWAS method effectively integrates individualized brain ROI spatial interactions to identify significant genetic variants, its focus on the brain limits the ability to capture causal SNPs with broader biological relevance. Other organs, such as the heart, kidneys, and liver, likely play significant roles in psychiatric disorders like BD, suggesting that incorporating multi-organ interactions could provide a more comprehensive understanding of the genetic foundations of disease. Extending Meta-sGWAS to a multi-organ framework requires constructing cross-organ interaction networks, treating each organ as a distinct entity while identifying disease-associated ROIs within each. Psychiatric and imaging data can guide the identification and validation of these cross-organ ROI networks, enabling the development of a systemic association map across organs. For example, correlations between heart rate, blood pressure, and neurological activity may reveal a brain-heart interaction, which could be clarified through Mendelian randomization to establish causal links between ROIs and disease. This approach would enhance the biological relevance and accuracy of GWAS results by uncovering key organ-specific ROIs and their interactions. Integrating these multi-organ interaction features into Meta-sGWAS would provide a comprehensive framework to capture systemic genetic influences across a whole-body scale, offering deeper insights into the functional changes underlying complex disorders like BD. Such a cross-organ approach not only enhances our understanding of systemic diseases but also lays the foundation for precision medicine targeting multi-organ interactions, enabling more effective interventions and providing valuable tools for future research.

## 4 Methods

### Study cohorts

The Meta-sGWAS included two datasets: the ABCD dataset and the UK Biobank (UKB) dataset. Whereas we use UKB as the external validation dataset for the findings in the ABCD dataset. For the meta-analysis part, we derived new and previously reported GWAS summary statistics of phenotype Bipolar Disorder from 40 studies (summarized in the supplementary S3 Table), the summary statistics are exported through GWAS Catalog.

The ABCD study stands as the preeminent long-term investigation into adolescent brain development and child health within the United States. Utilizing a comprehensive school-based recruitment approach, the ABCD study gathers neuroimaging, genetic, and clinical data from children aged 9–10 years old of different races across 21 research centers nationwide. We collected 9,345 samples with genetic data and brain imaging data from ABCD annual released 5.0 dataset. The details of the demographics are shown in Supplementary Fig. S5 a-d.

The UK Biobank (UKB) is one of the largest biomedical databases with genetic and health data from over half a million participants aged 40–69 across the UK. For this study, we included a subset of 11,209 participants, including 116 cases and 11, 093 controls, with complete genetic and brain imaging data related to Bipolar Disorder. Although the samples in the UKB are mostly European, the cohort also includes individuals from diverse backgrounds (e.g. Aisan and Black), offering diversity to generalize the findings.

### Brain imaging data

The ABCD study employs a range of MRI modalities to comprehensively investigate brain structure and function in its participants. These modalities include diffusion MRI with both diffusion tensor imaging (dMRI-DTI) and diffusion kurtosis imaging (dMRI-RSI), functional MRI during resting state (fMRI-RS), and task-based activities (fMRI-task), as well as structural MRI (sMRI). These modalities allow for the examination of white matter microstructure, brain activity during both task engagement and rest, and detailed anatomical imaging. The UKB only contains structural MRI (sMRI).

Raw imaging data, including different types of sMRI, dMRI, and fMRI, are acquired from participants. Preprocessing steps include correction for motion artifacts, spatial normalization to a common template, and intensity normalization. For sMRI, this involves tissue segmentation with Freesurfer to measure gray matter, white matter, and cerebrospinal fluid. For dMRI, diffusion tensor imaging (DTI) metrics such as fractional anisotropy are computed to assess white matter microstructure. Resting-state fMRI data are processed to identify intrinsic connectivity networks using methods such as independent component analysis. Task-based fMRI data are analyzed to characterize brain activity patterns during specific cognitive tasks.

In this study, we categorized and mapped the preprocessed MRI data into 148 different ROIs.

### Genetic data quality control and imputation

The ABCD study collects saliva samples from participants for DNA extraction and subsequent genotyping using the Smokescreen genotyping array. Initially, the dataset consisted of 515, 279 genetic variants from 11, 666 individuals. After applying stringent quality control criteria, such as a minor allele frequency (MAF) threshold of 0.05, a Hardy-Weinberg equilibrium (HWE) threshold of 10^−6^, and the exclusion of samples with more than 20% missing data, the dataset was refined to include 276,345 variants across 9,345 subjects.

Similarly, in the UK Biobank (UKB) study, biological samples including blood, urine, and saliva were collected from participants recruited between 2006 and 2010. The initial genetic dataset contained 784, 256 variants across 488,377 participants. Following rigorous quality control, which involved the same MAF and HWE thresholds as in the ABCD study, along with the removal of samples with high missing data ratios, the final dataset comprised 4, 323, 322 variants from 487, 409 individuals.

Both datasets followed quality control procedures, genotype imputation was conducted using the GENome-wide Imputation PipelinE (genip) [25] tool, which integrates PLINK, SHAPEIT, and IMPUTE2 for the efficient imputation of missing genotype data. The reference panel utilized for imputation in this research was sourced from the genetic data of 1000 Genomes Phase 3. This approach utilizes a comprehensive reference dataset to infer missing genotypes, resulting in the imputation of genetic variants to a total of 13, 739, 993 for the ABCD and 93, 095, 623 for UKB. Subsequently, post-imputation quality control procedures were implemented to further refine the genotype data, resulting in the retention of 3, 948, 476 variants for ABCD and 4, 323, 322 for UKB.

### Data Simulation

To evaluate our model, we generated synthetic data using the PhenotypeSimulator tool [26], similar research also adopted this method such as Zeng et al. [27], which integrated genetic data with brain-specific expression profiles to identify causal variants associated with brain traits. PhenotypeSimulator simulates phenotypes within the framework of linear mixed models (LMMs) that account for genetic variant effects and non-genetic components across multiple traits, creating a robust framework for benchmarking. To model brain interaction patterns, we introduced individualized brain activity profiles by sampling from a normal distribution with a mean ($\phi_{i}$) and variance of 10, where $\phi_{i}$ represents each individual’s average ROI value. This configuration captures realistic brain variability, while generating $\phi_{i}$ values from a uniform distribution (0–1) and simulates phenotypic diversity. Finally, we calculated a 1,000-by-1,000 brain interaction correlation matrix using Pearson coefficients to mimic the brain interaction patterns. Heatmaps in Supplementary Fig. S1 visualize these individualized patterns and sample similarities, demonstrating the method’s alignment with advanced techniques for analyzing complex brain traits.

### Meta-sGWAS framework

#### Heritability Estimation

The genetic relationship matrix (GRM) was calculated using GCTA [28] from genome-wide single nucleotide polymorphism (SNP) data. The GRM quantifies the genetic similarity between pairs of individuals based on their genotypes. It is an essential component in the linear mixed model (LMM) used for heritability estimation. We first calculate the GRM from autosomal SNPs after quality control in the ABCD dataset. Then the heritability was estimated for various traits using the LMM approach, as implemented in GCTA.

The phenotype y of each individual was modeled using the linear mixed model:

(1) y=Xβ+Zu+ϵ

where y is an n×1 vector of measurement from 148 ROIs of different MRI modalities for n individuals, X is an n×p matrix of fixed effects (including intercept and covariates), β is a p×1 vector of fixed-effect coefficients, Z is an n×q matrix relating individuals to random effects, u is a q×1 vector of random effects, and ϵ is an n×1 vector of residuals. The random effects u are assumed to follow a multivariate normal distribution $𝒩\left(0,\sigma_{g}^{2}G\right)$, where $\sigma_{g}^{2}$ is the additive genetic variance and G is the GRM. The residuals ϵ are assumed to follow $𝒩\left(0,\sigma_{e}^{2}I\right)$, where $\sigma_{e}^{2}$ is the residual variance.

The variance of the phenotypic trait was decomposed into genetic and residual components:

(2) $$\text{Var}(y)=\sigma_{g}^{2}G+\sigma_{e}^{2}I$$

The narrow-sense heritability $h^{2}$ was estimated as:

(3) $$h^{2}=\frac{\sigma_{g}^{2}}{\sigma_{g}^{2}+\sigma_{e}^{2}}$$

For each trait, phenotypic data were extracted from CSV files, and the GRM was used as the random effect in the LMM. GCTA was run with the ‘−reml’ option to perform Restricted Maximum Likelihood (REML) estimation of variance components. The analysis was parallelized by trait, using 15 threads for each GCTA instance. The estimated heritability for each trait was saved to the output directory.

All analyses were performed using GCTA version 1.94.1, with computations carried out on a high-performance computing cluster. The GRM was constructed using the entire genotype data set available for each study subject, and phenotypic data were extracted from 5 different MRI modalities in the ABCD.

#### Mendelian Randomization

Mendelian randomization (MR) is a method that uses genetic instrumental variables (IVs) to assess the causal direction between exposure and outcome, and it is unaffected by confounding factors and reverse causality [29, 30]. In the face of clinical research where randomized controlled trials (RCTs) are challenging to conduct, including but not limited to impractical and unethical RCT studies, MR can strengthen the inference of direct causal relationships between exposure factors and diseases, while avoiding difficult RCT studies [29]. This study used a two-sample Mendelian randomization analysis to estimate the association between structural changes in different brain regions and BD.

Genetic correlation is a central parameter for understanding the shared genetic architecture between complex traits. Using summary statistics from genome-wide association studies (GWAS), linkage disequilibrium score regression (LDSC) can provide an unbiased estimation of genetic correlations. LDSC allows for the assessment of SNP-based genetic heritability correlations between two traits [31], we preliminarily applied LDSC to evaluate the genetic correlation between structural changes in different brain regions and BD, using European ancestry samples from the 1000 Genomes Project as the reference panel [32]. First, we calculated the genetic correlation between each brain region and BD, retaining only those with a P-value less than 0.05. For these filtered brain regions, we performed Mendelian randomization to examine the causal relationship between structural changes and BD.

The MR analysis was primarily performed using the IVW (Inverse Variance Weighted) approach, which assumes the absence of an average pleiotropic effect [33]. To evaluate heterogeneity introduced by different genetic variants, Cochran’s Q statistic was calculated under the fixed-effect IVW model. A P-value less than 0.05 indicates the presence of heterogeneity. To assess the robustness of the results, a leave-one-out (LOO) analysis was conducted to determine if any single SNP drove the overall effect. The statistical analyses were completely performed in R software (version 4.3.0, R Foundation for Statistical Computing) with ldscr (version 0.1.0) [31], TwoSampleMR (version 0.5.7) [34], and MendelianRandomization (version 0.8.0) packages. Effect estimates were reported in β values with 95% CI where the outcome was BD disease.

#### Significant genetic vatiants identification

Meta-sGWAS aims to identify significant Single Nucleotide Polymorphisms (SNPs) associated with neuropsychiatric-related diseases through the analysis of personalized brain spatial interaction patterns and genetic features. In this study, we generated individualized brain interaction maps by integrating brain imaging-derived phenotypes (IDPs) obtained from different MRI modalities data.

This innovative approach is developed based on generalized linear mixed models. Denote covariates as X (e.g., demographic data and principal components (PCs)), and genetic variants as G, are designated as fixed effects. The model not only estimates coefficients α and β for covariates and genetic variants, but will also estimate a random slope b(S) that contains information from pre-defined brain ROIs S, for samples from a variance-covariance matrix V(s), which encapsulates the similarities in brain patterns among the samples. So, the model can be formulated as g(Y)=Xα+Gβ+b(S)+ε, where g(⋅) is the link function, the random effect b(S) follows a multivariate normal distribution MVN(0,τV(S)), and τ is the variance component for the random effects to be estimated. We aim to analyze the P-values from the coefficients of genetic variants and identify the potentially significant ones. To construct the variance-covariance matrix V(S) for the brain interaction pattern similarity, it can be captured through a three-step process:

1. We first mapped different MRI modalities data to pre-defined ROIs. The data containing brain IDPs such as the volume, surface area, thickness, etc will be mapped to the pre-defined ROIs. So each brain ROI contains a vector of IDPs.
2. Then we performed association tests between the brain IDPs and the phenotype, filtering out insignificant IDPs that were deemed unrelated to the BD. And we construct the significant IDPs ($P<10^{-5}$) representing the pre-defined ROIs. For example, the features of brain ROI k in sample i can be defined as vector $s_{k}^{\left(i\right)}$.
3. Lastly, we construct the brain spatial kernel pattern using two components: the distances and the activities of the m ROIs. The distances are measured in Euclidean distance from the MNI coordinates of ROIs, and the activities are the normalized values of the most significant brain IDPs from various MRI data modalities (e.g., ABCD dataset contains dMRI-DTI, dMRI-RS, sMRI, fMRI-RS, and fMRI-task). A Gaussian Radial Basis Function (RBF) kernel, defined as $Ker\left(s_{k}^{\left(\cdot \right)},s_{l}^{\left(\cdot \right)}\right)=\text{exp}\left(-\frac{{\left\|s_{k}^{\left(\cdot \right)}-s_{l}^{\left(\cdot \right)}\right\|}^{2}}{2\sigma^{2}}\right)$, is employed to calculate the correlation between k-th ROI and l-th ROI within any sample (denote as ·). Thus, the individualized brain spatial kernel pattern $R\left(s^{\left(i\right)}\right)$ for the i-th sample is defined as $R\left(s^{\left(i\right)}\right)=Ker{\left(s_{k}^{\left(i\right)},s_{l}^{\left(i\right)}\right)}_{\left(k\neq l,\forall k,l\in (1,m)\right)}$.

The variance-covariance matrix V is a symmetric matrix, where $v_{ij}$ denotes the Pearson correlation between the i-th and j-th patients. Specifically, $v_{ij}=\text{corr}\left(R\left(s^{\left(i)\right)},R\left(s^{\left(j\right)}\right)\right)\right.$, where $\text{corr}\left(R\left(s^{\left(i\right)}\right),R\left(s^{\left(j\right)}\right)\right)$ represents the Pearson correlation between the individualized brain interaction patterns $R\left(s^{\left(i\right)}\right)$ and $R\left(s^{\left(j\right)}\right)$.

Meta-sGWAS takes the individualized brain interaction patterns as the random effects and the genetic variants as the fixed effects. The the probability of binary outcome can be defined as η=g(Xα+Gβ+b)=P(Y=1∣X,G,b), where b is denoted as b(s) for simplicity. Denote a variance function ν(⋅), and ν(η)=Var(y∣b). Then, the Quasi Likelihood function of sGWAS is of form

$$ql(\alpha ,\beta ,\tau )=\text{log}\int \prod \text{exp}(ql(\alpha ,\beta ,b(s);y))\times (2\pi {)}^{n/2}|\tau V{|}^{-1/2}\times \text{exp}\left\{-\frac{1}{2}b^{⊤}(\tau V{)}^{-1}b\right\}db$$

and with Pearson Chi-square approximation,

$$ql(\alpha ,\beta ,b;y)\approx \frac{(y-\eta {)}^{2}}{\nu (\eta )}$$

After obtaining the results from the previous step, Meta-sGWAS conducts a meta-analysis by integrating these results with other published GWAS findings on the same phenotypes. Here, we use the summary statistics from 40 studies to adjust for potential heterogeneity and combine the effects to derive more robust associations. We assume that the effect size is the same across all studies, the combined effect size ${\overset{ˆ}{\beta }}_{\text{meta}}$ can be estimated using a fixed-effects model. The weight for each study i is $w_{i}=1/\sigma_{i}^{2}$, where $\sigma_{i}^{2}$ is the variance of the effect size estimate from study i.

$${\overset{ˆ}{\beta }}_{\text{meta}}=\frac{\sum i=1^{k}w_{i}\beta_{i}}{\sum_{i=1}^{k}w_{i}}$$

The variance of the combined effect size is:

$$\text{Var}(\overset{ˆ}{\beta })=\frac{1}{\sum i=1^{k}w_{i}}$$

When there is heterogeneity among the studies, a random-effects model is more appropriate. The combined effect size $\overset{ˆ}{\beta }$ accounts for both within-study and between-study variance. The total variance is $\tau^{2}+\sigma_{i}^{2}$, where $\tau^{2}$ is the between-study variance.


$${\overset{ˆ}{\beta }}_{\text{meta}}=\frac{\sum_{i=1}^{k}w_{i}^{*}\beta_{i}}{\sum_{i=1}^{k}w_{i}}$$

where the weight $w_{i}^{=}1/\left(\tau^{2}+\sigma_{i}^{2}\right)$. The variance of the combined effect size is:

$$\text{Var}\left({\overset{ˆ}{\beta }}_{\text{meta}}\right)=\frac{1}{\sum i=1^{k}w_{i}^{*}}$$

To estimate between-study variance ($\tau^{2}$), the DerSimonian and Laird (1986) [35] method is commonly used:

$$\tau_{\text{meta}}^{2}=\text{max}\left\{0,\frac{Q-(k-1)}{\sum_{i=1}^{k}w_{i}-\frac{\sum_{i=1}^{k}w_{i}^{2}}{\sum_{i=1}^{k}w_{i}}}\right\}$$

where Q is the Cochran’s Q statistic, defined as $Q=\sum_{i=1}^{k}w_{i}{\left(\beta_{i}-{\overset{ˆ}{\beta }}_{\text{meta}}\right)}^{2}$.

#### Verify significant SNPs in single-cell data

We implemented Single-Cell Disease Relevance Score (scDRS) to analyze downstream gene expressions in different cell types. scDRS integrates GWAS and MAGMA (Multi-marker Analysis of GenoMic Annotation) to identify disease-related cell types using single-cell RNA sequencing data[36]. The process begins by acquiring GWAS data, which includes SNPs’ p-values and effect sizes, to determine their association with the disease. MAGMA maps these significant SNPs to genes based on their genomic positions and performs gene set enrichment analysis to identify gene sets enriched in the disease[37]. scDRS scores are calculated by combining these gene expression levels with MAGMA results, weighting each gene’s expression by its significance derived from GWAS data. Specifically, scDRS links SNPs to genes through their positions and associations, genes to cells through their expression profiles, and cells to disease by evaluating the cumulative impact of disease-associated genes on individual cells. Dimensionality reduction techniques like UMAP or t-SNE visualize the distribution of scDRS scores across cell types, highlighting those significantly associated with the disease. This method enables the identification of disease-related cells and cell types, enhancing the understanding of disease mechanisms at the cellular and molecular levels.

## Supplementary Material

This is a list of supplementary files associated with this preprint. Click to download.

- heritabilityS1.csv
- SharedSNPsS2.csv
- metaanalysisdescriptionS3.csv
- SupplementaryFigures.pdf
- SupplementaryTableS4.xlsx

**Supplementary information.** The paper has three supplementary figures and four tables.

## Data Availability

ABCD and UKB are open-source databases with restrictions.

## Appendix A Figures

- **S1 Fig. Synthetic data example.** a) Synthetic brain ROI data in 20 randomly selected samples in 10 ROIs; b) 10 randomly selected synthetic samples correlation.
- **S2 Fig. Hierarchical heatmap of brain ROIs.** Brain interactions among the 148 ROIs in 5 different MRI modalities, the hierarchical clustering is based on the mean of MRI value in z-scores t-test.
- **S3 Fig. Example of brain interactions and correlation heatmap.** a) Example of brain interactions from BD-sample and non-BD sample; b) Correlation heatmap from 20 randomly selected samples in the ABCD dataset.
- **S4 Fig. Network diagram between SNPs and psychiatric disorder.** Network diagram illustrating relationships between SNPs and psychiatric disorders.
- **S5 Fig. Demographic of ABCD and UKB data.** a) Age distribution of ABCD dataset with BD; b) Race distribution of ABCD dataset with BD; c) Age distribution of UKB dataset with BD; d) Race distribution of UKB dataset with BD.
- **S6 Fig. SNP rs113779084 single-cell analysis.** Single-cell analysis of SNP rs113779084.

## Appendix B Tables

- **S1 Table. Heritability analysis results.** Results of heritability analysis.
- **S2 Table. Shared significant SNPs results.** Results of shared significant SNPs analysis.
- **S3 Table. Summary of meta-analysis studies used in this study.** Summary of meta-analysis studies utilized in this study.
- **S4 Table. Summary of identified significant SNPs in other psychiatric disorders.** Summary of identified significant SNPs associated with other psychiatric disorders.
